# The role of oxygen depletion and subsequent radioprotective effects during irradiation of mosquito pupae in water

**DOI:** 10.1186/s13071-020-04069-3

**Published:** 2020-04-17

**Authors:** Hanano Yamada, Hamidou Maiga, Nanwintoum Severin Bimbile-Somda, Danilo O. Carvalho, Wadaka Mamai, Carina Kraupa, Andrew G. Parker, Aiman Abrahim, Georg Weltin, Thomas Wallner, Marc F. Schetelig, Carlos Caceres, Jeremy Bouyer

**Affiliations:** 1 Insect Pest Control Laboratory, Joint FAO/IAEA Division of Nuclear Techniques in Food and Agriculture, International Atomic Energy Agency, Vienna International Centre, P.O. Box 100, 1400 Vienna, Austria; 2 Food and Environmental Protection Laboratory, Joint FAO/IAEA Division of Nuclear Techniques in Food and Agriculture, International Atomic Energy Agency, Vienna International Centre, P.O. Box 100, 1400 Vienna, Austria; 3 Soil and Water Management & Crop Nutrition Laboratory, Joint FAO/IAEA Division of Nuclear Techniques in Food and Agriculture, International Atomic Energy Agency, Vienna International Centre, P.O. Box 100, 1400 Vienna, Austria; 4 Institute for Insect Biotechnology, Department of Insect Biotechnology in Plant Protection, Justus-Liebig-University Gießen, Winchester Str. 2, 35394 Giessen, Germany

**Keywords:** *Aedes aegypti*, *Aedes albopictus*, *Anopheles arabiensis*, Dissolved oxygen, Pupa respiration, Hypoxia, Irradiation, Gammacell, Induced sterility, SIT

## Abstract

**Background:**

Radiation induced sterility is the basis of the Sterile Insect Technique, by which a target insect pest population is suppressed by releasing artificially reared sterile males of the pest species in overflooding numbers over a target site. In order for the sterile males to be of high biological quality, effective standard irradiation protocols are required. Following studies investigating the effects of mosquito pupae irradiation in water *versus* in air, there is a need to investigate the oxy-regulatory behavior of mosquito pupae in water to better understand the consequences of irradiation in hypoxic versus normoxic conditions.

**Methods:**

Pupae of *Aedes aegypti*, *Ae. albopictus*, and *Anopheles arabiensis* were submerged in water inside air-tight 2 ml glass vials at a density of 100 pupae/ml and the dissolved oxygen (DO) levels in the water were measured and plotted over time. In addition, male pupae of *Ae. aegypti* (aged 40–44 h), *Ae. albopictus* (aged 40–44 h) and *An. arabiensis* (aged 20–24 h) were irradiated in a gammacell220 at increasing doses in either hypoxic (water with < 0.5% O_2_ content) or normoxic (in air) conditions. The males were then mated to virgin females and resulting eggs were checked for induced sterility.

**Results:**

All three species depleted the water of DO to levels under 0.5% within 30 minutes, with *An. arabiensis* consuming oxygen the fastest at under 10 minutes. Following irradiation, the protective effect of hypoxia was observed across species and doses (*P* < 0.0001), increasing at higher doses. This effect was most pronounced in *An. arabiensis*.

**Conclusions:**

The consumption of dissolved oxygen by pupae submerged in water was significantly different between species, indicating that their oxy-regulatory capacity seems to have possibly evolved according to their preferred breeding site characteristics. This needs to be considered when sterilizing male mosquitoes at pupal stage in water. Depending on species, their DO consumption rates and their density, irradiation doses needed to achieve full sterility may vary significantly. Further assessments are required to ascertain optimal conditions in terms of ambient atmosphere during pupal irradiation to produce competitive sterile males, and temperature and density dependent effects are expected.
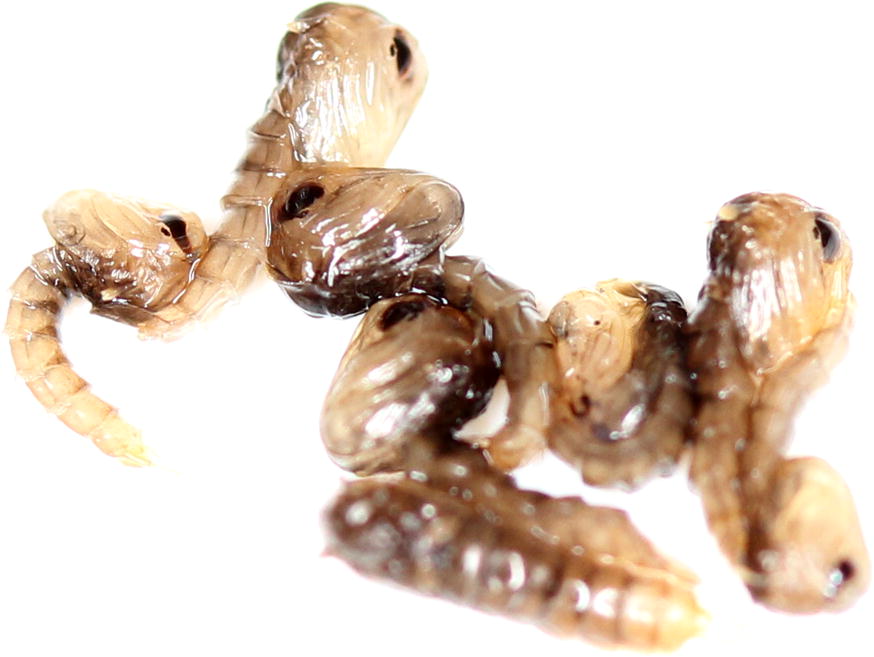

## Background

The management of mosquitoes (and other insect pests) using the sterile insect technique (SIT) relies on the successful mating of factory reared, sexually sterilized males that are released into the target area in overflooding numbers with wild females [1]. The SIT has been implemented as a part of Area-Wide Integrated Pest Management (AW-IPM) programmes for a variety of insect pests over the past 60 years, and has also been assessed for the suppression of some mosquito species, notably *Anopheles albimanus* [2, 3] and several *Culex* species [4–9], in the 1970s and 1980s. Only recently, the development of the SIT for the management of other mosquito species has begun to gain more focus in response to outbreaks of chikungunya in Europe, global increasing incidences of dengue fever, the re-emergence of yellow fever and the recent emergence of the Zika virus, which are all transmitted by *Aedes aegypti* and/or *Aedes albopictus* [10–17].

There are several options to achieve sterility in insects [18] including transgenic approaches [19], use of *Wolbachia* (i.e. for mating incompatibility) [20], use of chemicals and use of ionizing radiation [21]. Historically, a variety of chemosterilants have been used for the reproductive sterilization of male mosquitoes with varying success and suitability for larger scale SIT programs [3, 9, 11, 22]. However, the evaluation of sterilizing male mosquitoes by irradiation has suggested that this is, to date, the most practical (and environmentally friendly) way to induce sterility, especially at large scale [23]. Some publications reporting the dose-response of mosquitoes are available [23–26], and more recently, more work has been done regarding the effects of irradiation on mosquito fertility, longevity, flight ability and mating competitiveness [27–33] providing a good basis for the SIT package for mosquitoes. However, a more detailed look into other factors affecting radiation induced sterility, including handling methods, irradiation device and source, and intrinsic biological factors of the mosquito, are essential for the development and formulation of standard protocols for the reliable and reproducible induction of sterility.

One of the main factors known to change the radiosensitivity in insects is the atmospheric conditions that they are subjected to before, and during radiation exposure. In Mediterranean fruit flies (*Ceratitis capitata*), it has been shown that the conditioning of the pupae pre-irradiation by inducing hypoxic conditions affected the radiosensitivity of the flies and reduced the “oxygen effects” during irradiation, resulting in more competitive sterile males [34]. Irradiating agricultural commodities in the context of phytosanitary treatments in reduced levels of oxygen rendered other insect species such as the apple maggot (*Rhagoletis pomonella*), oriental fruit moths (*Grapholita molesta*), European corn borer (*Ostrinia nubilalis*), and plum curculio (*Conotrachelus nenuphar*) more resistant to the radiation treatment and residual fertility in some cases increased up to 20-fold *versus* those irradiated in air [35–37].

Whilst protocols for the irradiation of agricultural pests have become very standardized following decades of experience in the context of the SIT and phytosanitary treatments [38], mosquito irradiation protocols around the world are still very diverse, making reliability and reproducibility difficult. As male pupae sterilization is shifting from small sample sizes in Petri dishes for experimental work to bulk exposures of thousands or hundreds of thousands for field releases, handling and exposure protocols are changing for improved efficiency and practicality. A change in dose required for the complete sterilization of *Ae. albopictus* pupae was seen following bulk irradiation in water [39, 40], suggesting that the respiration or transpiration of the pupae reduces dissolved oxygen levels in the water, thus creating a hypoxic environment. However, little has been demonstrated in mosquito pupae in terms of their mechanism(s) of oxygen usage and their behavior when fully submerged under water. Generally, mosquito pupae seem to be bimodal breathers, obtaining oxygen *via* their respiratory trumpets directly from the air when they float on the water surface [41], and *via* diffusion of dissolved oxygen from the water through their cuticle when submerged [42, 43]. Aquatic insects often have a thin permeable integument that enables cuticular respiration and thus the diffusion of gases while under water.

To evaluate the oxygen consumption characteristics of mosquito pupae for irradiation purposes, we assessed dissolved oxygen depletion by pupae in small artificial aqueous environments to ensure the time required to achieve hypoxic conditions prior to evaluating the impact of hypoxia on the dose-response to irradiation for *Ae. aegypti*, *Ae. albopictus* and *An. arabiensis* male pupae. The resulting data aim to improve protocols for the SIT against these mosquito species.

## Methods

### Mosquito strains

The *Ae. albopictus* strain used for the experiment originated from field collections in northern Italy and has been maintained at the Centro Agricoltura Ambiente, Bologna, Italy. The strain was transferred to the Insect Pest Control Laboratory (IPCL) of the FAO/IAEA Agriculture & Biotechnology Laboratories, Seibersdorf, Austria in 2010.

The *Ae. aegypti* strain originated from field collections in Juazeiro (Bahia), Brazil and was transferred to the ICPL from the insectary of Biofabrica Moscamed, Juazeiro, Brazil, in 2016. Both the *Aedes* strains have been maintained following the “Guidelines for Routine Colony Maintenance of *Aedes* mosquitoes” [44].

The Dongola strain of *An. arabiensis*, originating from Dongola, Northern State, Sudan, was donated by the Tropical Medical Research Institute, Khartoum, Sudan, in 2010 and has been maintained at the IPCL following the anopheline mass-rearing guidelines [45].

### Measurement of oxygen depletion by pupae in water

Pupae from the first day of pupation (and therefore mostly male) of each mosquito species were counted into batches of 200 and were transferred into 2 ml autosampler vials (Merck KGaA, Darmstadt, Germany) which were then topped up to the rim with water to ensure that there is no air bubble inside the vial when closed. The water and ambient temperature were measured at 25 °C. The temperature and dissolved oxygen (DO) in the water was measured with a DO-166MT-1 micro dissolved Oxygen electrode (Lazar Research Laboratories, Inc., Los Angeles, CA, USA) which was inserted into the sample through the hole in the cap of the autosampler vial and was fixed by a plastic valve and blu tac adhesive putty. The electrode was attached to an amplifier and then to a Microcomputer based pH/mV/Temp portable meter (Jenco Model 6230N, Jenco Instruments Inc., San Diego, CA, USA). The electrode was calibrated prior to each experiment using a two-point calibration: in air (21% oxygen, and in water which was bubbled with Nitrogen for 2 h to obtain a 0% DO reference point, as described by Butler et al. [46]. The DO in the sample was measured in 1-second intervals and data were recorded and plotted using the ArrowDO software provided by Lazar Research Laboratories and Microsoft Excel v16.0 (Microsoft, 216 Redmond, WA. USA). Dissolved oxygen curves were compared between species. There were at least 3 biological repetitions for each species, i.e. batches were derived from different cohorts and were tested on different days. Each cohort also included between 2 and 6 technical repetitions with different batches of 200 pupae that were derived from the same cohort.

### Irradiation of pupae in hypoxia *versus* normoxia

Pupae of all three species were collected in 4-h windows to ensure uniform pupal age of 40–44 hours for both *Aedes* species, and 20–24 hours for *An. arabiensis*. We chose these age groups as these represent the last hours before they begin to emerge into adults (*An. arabiensis* has a much shorter pupal duration than *Aedes* spp.). *Aedes* pupae were sexed based on pupal size dimorphism using a glass pupal sorter [47] and sex was verified under a stereomicroscope. Pupae of *An. arabiensis* were sexed visually using a stereomicroscope. Males were kept for treatment and females were placed in individual tubes for emergence to ensure virginity for later mating. Male pupae were counted into batches of 80 and were placed either in 0.5 ml Eppendorf tubes in water (for the hypoxic conditions) or into the center (2 cm diameter ring made with hot-melt adhesive) of standard 100 mm × 15 mm Petri dishes (for normoxic (21% O_2_) conditions) for irradiation. Higher densities of pupae were used to ensure hypoxic conditions of < 0.5% DO after 30 min in all three species, as was seen in the previous experiment. The Eppendorf tubes were then closed 30 min prior to irradiation treatment to ensure hypoxia (< 0.5% DO) within the tubes. The pupae in both the hypoxic and normoxic treatment groups were irradiated at the same time. At least three technical repetitions and three biological repetitions were performed for all doses in all species.

Radiation treatments were performed in a Gammacell 220 (Nordion Ltd, Kanata, Ontario, Canada), with a current dose-rate of 84 Gy/min. The dose uniformity ratio within the volume used for the irradiators was 1.05 or less. Treatments and controls were performed in either hypoxic or normoxic conditions. The doses used were selected according to the expected dose required to induce 50–100% sterility in each strain: 20, 55, 70, 90 and 100 Gy for *Ae. aegypti*; 20, 35, 55 and 70 Gy for *Ae. albopictus*; 40, 75, 90, 110 and 120 Gy for *An. arabiensis* respectively.

### Dosimetry

The dosimetry system used to verify the dose received by the batches was based on Gafchromic HD-V2 and MD-V3 film (Ashland Advanced Materials, Bridgewater NJ, USA) following the protocol of the IAEA [48]. Three films of either HD film (for doses > 50 Gy) or MD film (for doses < 50 Gy) were packed in aluminium envelopes (to avoid getting wet) and placed directly above and below the pupae samples. The temperature near the sample and films was measured before and after radiation exposure. Films were read with an optical density reader after 24 h of development.

### Assessment of induced sterility

Following irradiation, 50 males were randomly selected from each treatment group and were placed in a 15 × 15 × 15 cm Bugdorm^®^ cage (MegaView Science Co. Ltd., Taichung 40762, Taiwan) for emergence. Fifty virgin females were added to each cage when the adults reached 2 days of age, and were allowed to mate for 3 days before they were provided with 2 blood meals on consecutive days (days 6 and 7 post-emergence). Oviposition cups containing water and germination papers (*Aedes* spp.) or filter papers (*Anopheles*) were added to each cage on day 8 for *en masse* egg collection (on days 9 and 10 post-emergence) following routine rearing protocols [44]. Egg papers from *Ae. aegypti* and *Ae. albopictus* were collected, matured (slow-dried over 4 days) and stored for 10 days before hatching. The *An. arabiensis* eggs were collected and hatched the same day. The total number of eggs and the number of first-instar larvae (L1) were counted for each treatment group to derive the hatch rate which was determined by counting the number of hatched and un-hatched eggs using a stereomicroscope. The residual fertility was calculated as a percentage of the control fertility [49]. Induced sterility (IS) was calculated by subtracting the RF from 100%. For overall results for each mosquito strain, all data from the biological and technical repetitions were pooled to attain the mean IS, and the median and upper and lower quartiles shown in the figures.

### Statistics

The dynamics of the O_2_ water concentration was assessed using a mixed effect binomial model, with the time, mosquito species and their first order interaction as fixed effects and the repeats as a random effect using the *lme4* package in R [50]. The response variable was either the raw O_2_ water concentration or its log-transform (log(c + 1)). The best model was identified using the corrected Akaikeʼs criterion (AICc) [51].

Binomial linear mixed effect models were used to analyze the impact of hypoxia and irradiation on the hatch rate (HR). The treatment regimen, the radiation dose and their first order interaction were used as fixed effects and the repetitions as random effects. The best model was selected on the basis of the lowest corrected Akaikeʼs information criterion (AICc), and the significance of fixed effects was tested using the likelihood ratio test [52]. All data were analyzed using the R language version 3.2.1 [53].

## Results

### Measurement of oxygen depletion by pupae in water

**Fig. 1:**
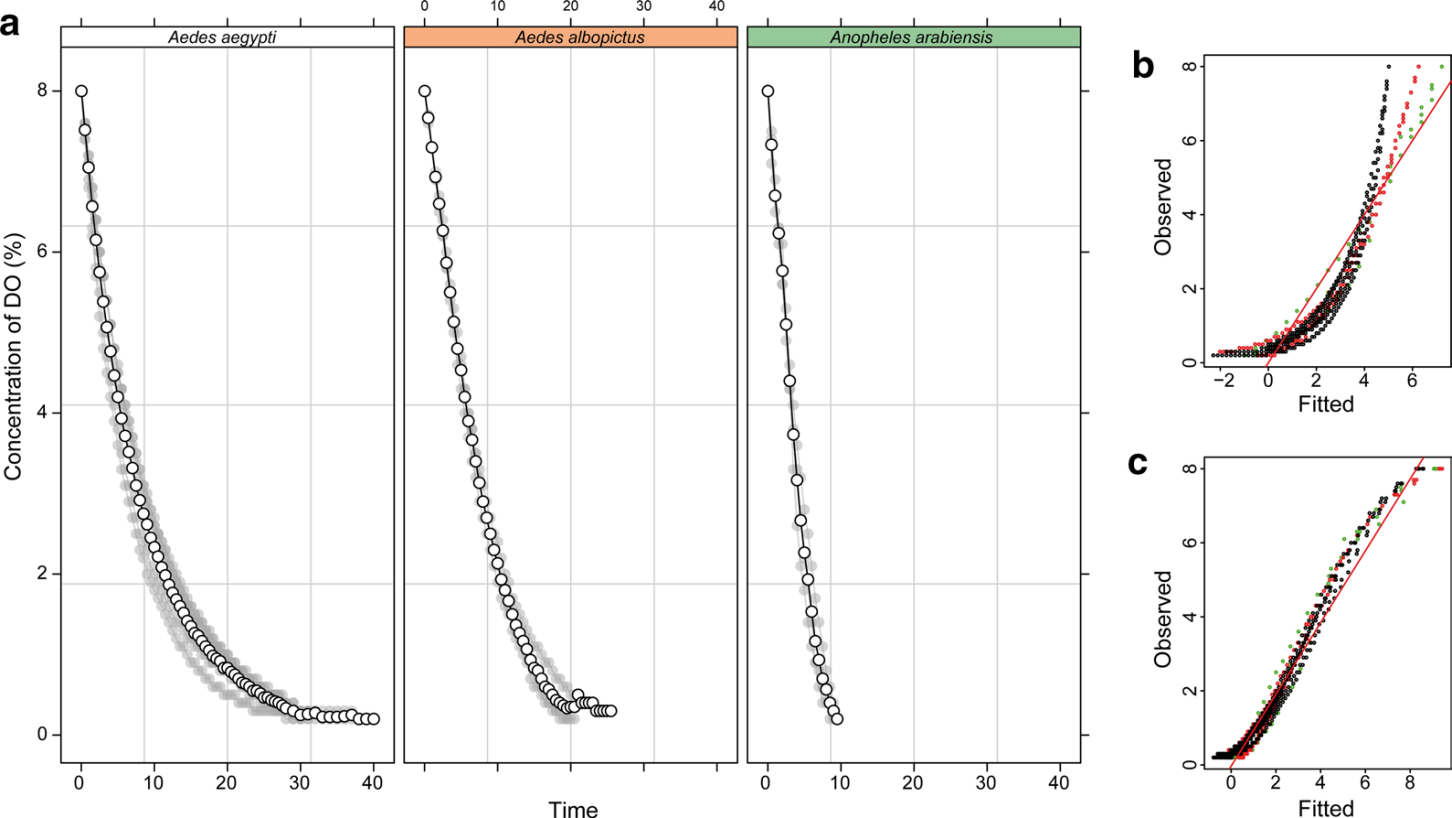
**a** Dissolved oxygen uptake by *Aedes aegypti*, *Aedes albopictus* and *Anopheles arabiensis* submerged in water. Dissolved oxygen (DO) level in percent (%) over time (minutes). **b** Raw O_2_ concentration. **c** Log-transformed O_2_ concentration (log(c + 1)). *Key*: black dots, *Ae. aegypti*; red dots, *Ae. albopictus*; green dots, *An. arabiensis*

Data on oxygen depletion by pupae in water are presented in Fig. 1. The three tested species differed significantly in their response to a closed aqueous environment. *Anopheles arabiensis* depleted the water of DO the fastest with levels plummeting to below 0.1% in under 10 min. The aedine pupae both required longer times to reach equally low DO levels, with *Ae. albopictus* reaching 0.1% DO in between 20–25 min, and *Ae. aegypti* requiring 30–40 min to reach DO levels under 0.3%. All pupae batches (all repetitions and all species) depleted DO levels to under 0.5% within 30 min, and thus 30 min was used to ensure this level of hypoxia for the following irradiation experiments.

The best statistical model to compare the three species responses was the full model with all fixed effects and using the log-transformed O_2_ concentration. However, the log-transformation improved the fit much more for *Aedes* species than in *Anopheles*, showing that the trend of DO reduction was much more linear in *An. arabiensis* which is also clear in Fig. 1, while the trend in both aedine species was non-linear. The DO content dropped significantly faster in *An. arabiensis* than *Ae. albopictus* (*P* < 0.0001) and again significantly faster in *Ae. albopictus* than *Ae. aegypti* (*P* < 0.0001).

### Irradiation of pupae in hypoxia *versus* normoxia

#### Dosimetry

The dosimetry confirmed that all doses received were within the 5% confidence interval of the dosimetry calibration.

#### Dose-response *Ae. aegypti*

**Fig. 2:**
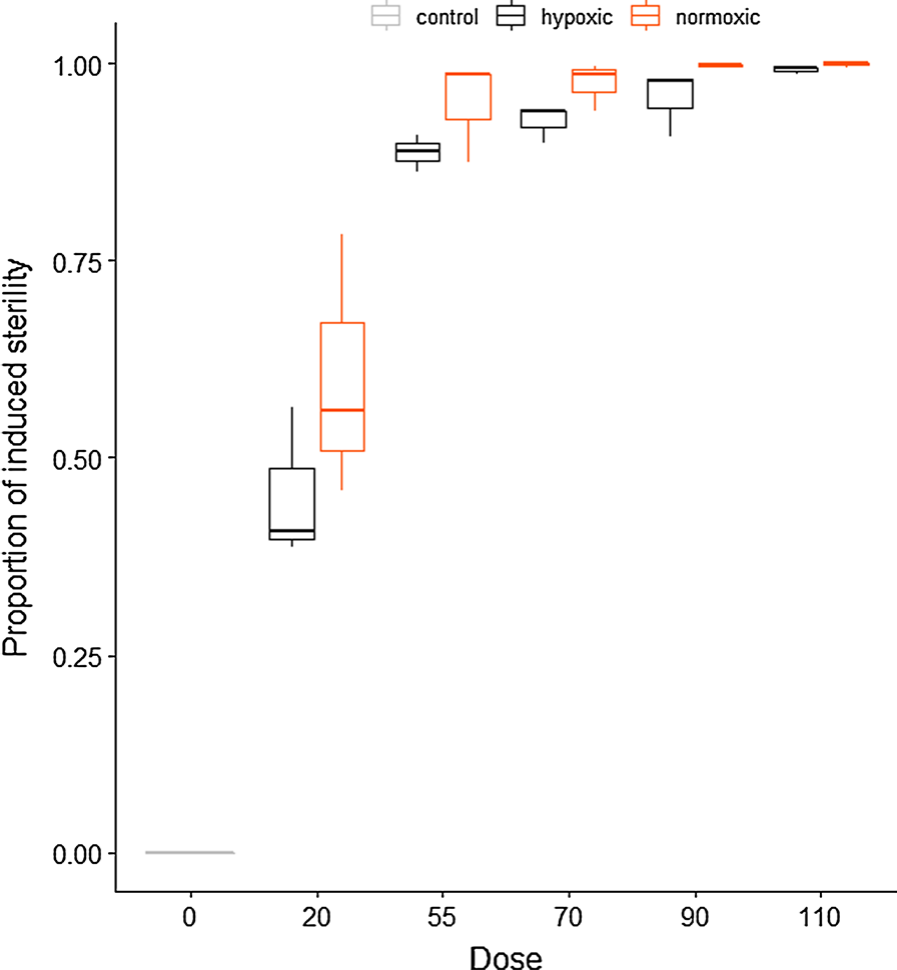
Dose-response curves for *Aedes aegypti* male pupae following irradiation in normoxic and hypoxic conditions. The box plot shows the median and upper and lower quartiles

Mean induced sterility (IS) for this species spanned from 60% at 20 Gy, to 100% at 90 Gy (and 110 Gy) following exposures in normoxic conditions. Mean induced sterility (IS) decreased significantly when irradiated in hypoxic conditions (45% to 99%) at the same doses (Fig. 2).

The best model included all fixed effects and the interaction between oxygen concentration and radiation dose. The hatch rate reduced with increasing dose (*P* < 0.0001, Table 1), but hypoxia only had a marginally “protective effect” against irradiation at lower doses, (*P* = 0.05). However, this protective effect increased significantly with dose (*P* < 0.0001).

**Table 1:** Fixed-effects coefficients of a mixed-effect Gaussian model of the impact of radiation dose on the hatch rate in *Aedes aegypti*

Fixed effects	Value	SE	*z*-value	*P*-value
Intercept	0.490346	0.076284	6.43	1.29e−10***
Hypoxia	0.151128	0.078640	1.92	0.0546
Dose	− 0.067274	0.001812	− 37.12	< 2e−16***
Hypoxia*Dose	0.020379	0.002037	10.00	< 2e−16***

***P ≤ 0.001

*Abbreviation*: SE, standard error

#### Dose-response *Ae. albopictus*

Mean induced sterility for this species ranged from 74% at 20 Gy, to 99.7% at 70 Gy following irradiation in normoxic conditions, compared to 21% IS and 84% in hypoxic conditions at the same doses.

**Fig. 3:**
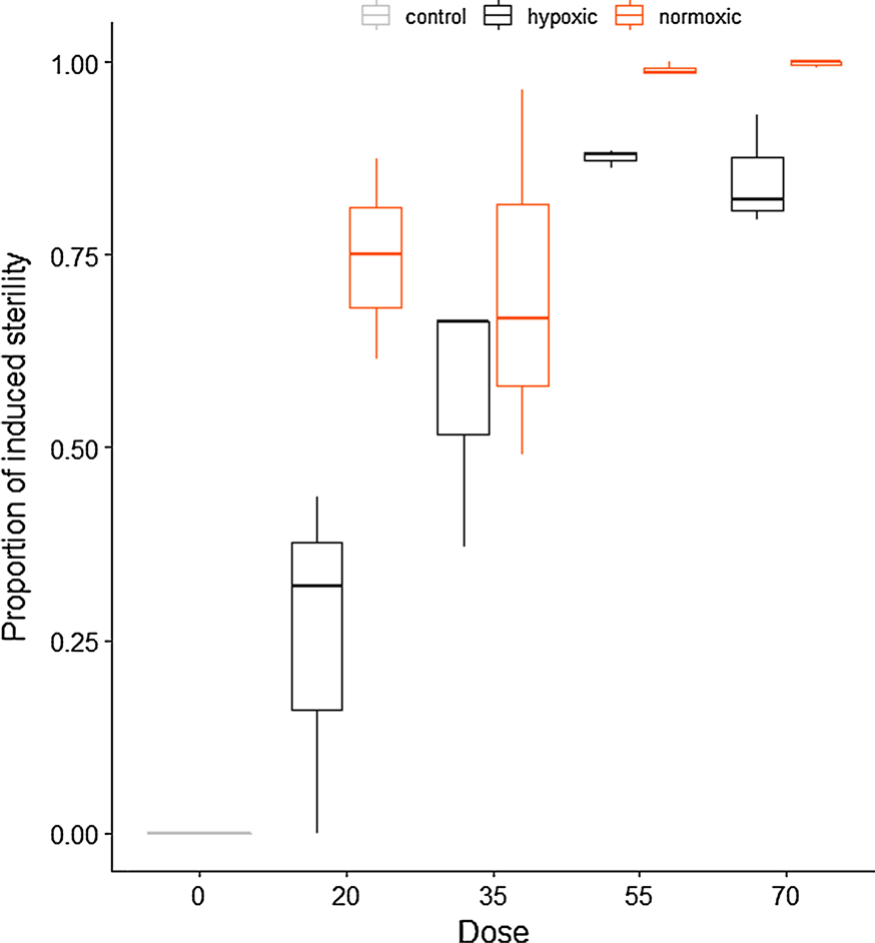
Dose-response curves for *Aedes albopictus* male pupae following irradiation in normoxic and hypoxic conditions. The box plot shows the median and upper and lower quartiles

The best model included all fixed effects and the interaction between oxygen concentration and radiation dose. The hatch rate reduced with increasing dose (*P* < 0.0001, Table 2), but hypoxia had a “protective effect” against irradiation (*P* < 0.0001). This protective effect increased with dose (*P* = 0.02). Hypoxia alone did not change the hatch rate in the control groups (*P* = 0.77) (Fig. 3).

**Table 2:** Fixed-effects coefficients of a mixed-effect binomial model of the impact of radiation dose on the hatch rate in *Aedes albopictus*

Fixed effects	Value	SE	*z*-value	*P*-value
Intercept	− 0.290276	0.249733	1.162	0.2451
Hypoxia	0.889215	0.205254	4.332	1.48e−05***
Dose	− 0.068176	0.006287	− 10.845	< 2e−16***
Hypoxia*Dose	0.014788	0.006650	2.224	0.0262*

*P ≤ 0.05; ***P ≤ 0.001

*Abbreviation*: SE, standard error

#### Dose-response *An. arabiensis*

**Fig. 4:**
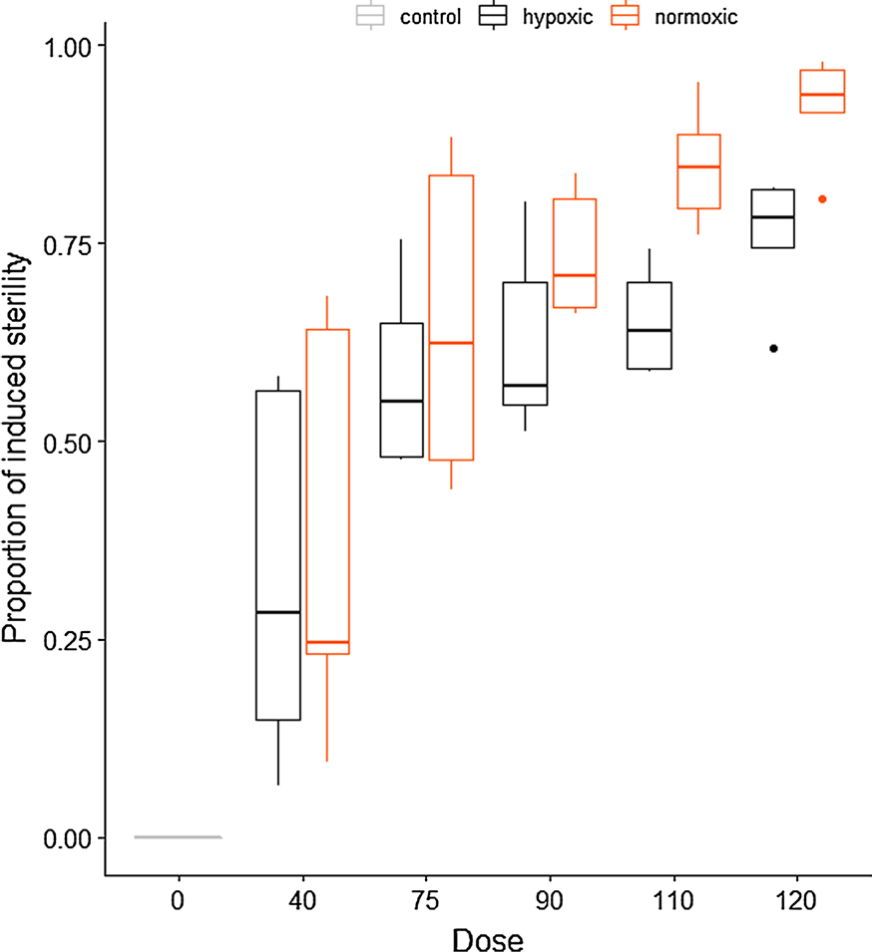
Dose-response curves for *Anopheles arabiensis* male pupae following irradiation in normoxic and hypoxic conditions. The box plot shows the median and upper and lower quartiles

The difference in mean induced sterility for this species when comparing results following irradiation in normoxic versus hypoxic conditions was less pronounced at the lowest doses tested (38% IS at 40 Gy in normoxia *versus* 33% IS at the same dose in hypoxia) but most pronounced of all three species tested at the highest dose of 120 Gy (92% IS in normoxia *versus* 76% IS in hypoxia), (Fig. 4).

The best model included all fixed effects and the interaction between oxygen concentration and radiation dose (Table 3).

**Table 3:** Fixed-effects coefficients of a mixed-effect Gaussian model of the impact of radiation dose on the hatch rate in *Anopheles arabiensis*

Fixed effects	Value	SE	z-value	*P*-value
Intercept	0.825774	0.206693	3.995	6.46e−05***
Hypoxia	− 0.476885	0.128439	− 3.713	0.000205***
Dose	− 0.028555	0.001238	− 23.064	< 2e−16***
Hypoxia*Dose	0.013450	0.001578	8.522	< 2e−16***

***P ≤ 0.001

*Abbreviation*: SE, standard error

The hatch rate reduced with increasing dose (*P* < 0.0001, Table 3). The protective effect of hypoxia against irradiation increased with the dose (*P* < 0.0001) and was even not observed at 40 Gy (leading to an artificial negative effect of hypoxia in the model that cannot be read without accounting for the interaction). Hypoxia alone did not change the hatch rate in the control groups (*P* = 0.98).

## Discussion

The oxygen depletion by pupae when submerged in water was clearly demonstrated in our study. It was surprising to see how differently the pupae of the three tested species coped with submergence in water and how quickly the DO was consumed from the surrounding water. However, after reviewing available literature on aquatic organisms and the evolutionary, behavioral and anatomical differences of the three species, the respiratory differences become more evident.

In terms of respiratory capabilities, animals can be classified as oxy-regulators and oxy-conformers, depending on their ability to respond to hypoxic conditions [54]. The oxygen consumption rates of oxy-conformers depends on the surrounding oxygen levels, i.e. they demonstrate a respiration rate proportional to the surrounding oxygen tension, whereas in contrast, oxy-regulators maintain their oxygen consumption rates independently of oxygen levels, until a critical oxygen tension is reached (a threshold level) at which point they can possibly convert into conformers [55]. Considering the results in this study, it is proposed that *An. arabiensis* (and probably all similar species) are oxy-regulators, as their respiration rates maintain constant despite DO levels decreasing around them, producing a linear response, while both *Ae. albopictus* and *Ae. aegypti* appear to be oxy-conformers as they seem to be able to downregulate respiratory activities as DO levels start to decrease in their surrounding environment, which is reflected by the non-linear response.

Our findings are supported by other biological differences described in available literature that are relevant to oxygen usage and respiratory activities for mosquitoes. Difference in these mosquito species are also reflected in their diving behavior. Mosquito pupae also use gas in their ventral air space (VAS) to be buoyant, and they lose this buoyancy when submerged under water [56]. *Culex pipiens* and *An. stephensi* are, for example, positively buoyant and typically make shallow, and short-duration dives, while many aedine species (for example *Ae. aegypti*, *Ae. albopictus* and *Ae. triseriatus*) tend to make deeper, and longer duration dives [56]. Although diving generally serves to escape predation, the enhanced diving capabilities in some aedine species is suggested to help keep the pupae from being washed out of their breeding container, or other shallow habitat [56].

The environmental history of insects may be an important factor that shapes the response to exposures in hypoxia [57]. The differences in tolerance to low oxygen environments may well have been an evolutionary response for some aedine species and now reflect their preferred small, shallow breeding sites often containing organic pollutants and microbial depletion of DO, whereas this adaptation, historically, was not necessary for the anopheline species which often select cleaner, larger bodies of water for breeding. However, in the recent decades, more and more reports describe the adaptation of *An. arabiensis* to a wider variety of breeding sites, including populations that have adapted to more polluted, urban habitats [58]. The strain of *An. arabiensis* used in this study was originally collected from larger, clean water bodies in Dongola, Norther Sudan before 2010. Preferred larval breeding sites included riverbanks, grassy knolls (flooded soil terraces near to the river), ‘khors’ (seasonal tributary channels), flood pools and wells [59]. It would be interesting to assess the tolerance to low oxygen environments in *An. arabiensis* populations collected more recently, from urban, shallow, and more polluted larval breeding sites to test the hypothesis that oxy-regulatory capacity evolved based on environmental factors in larval habitats. This physical adaptation and higher tolerance of some *Aedes spp*. to low oxygen environments- and thus their ability to stay submerged for longer periods may be supported by the structure of their tracheal branches which have been found to be different (and significantly larger), for instance, in *Ae. togoi* compared to *An. sinensis* [41].

Understanding the respiratory behavior in mosquito pupae when developing protocols for pupal irradiation, especially in large numbers, and the effects of low oxygen environments on dose-response is important as it is clear that dense pupae samples in water can create a hypoxic environment which provides radio-protection. This means that the dose used to induce the target sterility in small batches of pupae in air, is likely not sufficient for larger, more dense batches of pupae in water, and this could result in the release of sub-sterile males during a SIT programme. In addition to this, it is also important to note that oxygen solubility changes with temperature (DO increases as water temperature decreases), as does the metabolic rate of living organisms (generally metabolism decreases as temperature decreases). Therefore, the rate at which DO becomes depleted may change at lower temperatures. Interestingly, in some aquatic insect species (in the case of *Ilyocoris cimicoides*), only aerial respiration changed with temperature, while aquatic respiration did not [60]. This still needs further evaluation to better understand submerged pupal respiratory behavior in varying temperatures and hypoxia.

Following the assessment of DO depletion by pupae in water in the present study, and the results showing how quickly this occurs, a possible reason for varying sterility results following irradiation of pupae in water *versus* air, and in small batches *versus* large numbers [39, 40], becomes apparent. Thus, a reassessment of the dose-response in pupae was needed for both hypoxic and normoxic environmental conditions during irradiation treatments with the aim of standardizing irradiation protocols for mosquito pupae sterilization.

Most of the DNA damage produced by ionizing radiation comes from free radicals generated during the radiolysis of water, producing DNA strand breaks and other types of lesions that can be cytotoxic or mutagenic. Free radical-induced DNA damage is repaired by an efficient process involving several proteins [61]. These free radicals play an important role once they activate the processes of DNA repair. Therefore, initiating this process prior to radiation exposure could provide an adaptive resistance during irradiation. A study exposing insect cell cultures to X-rays found that, in these cells, the DNA damage and resistance to irradiation (in terms of survival) were greater than that of mammalian cells [62]. The authors’ assumption is that the abundance of free amino acids in the haemolymph can play a role in protecting the cell and supporting the repair machinery.

Irradiation in nitrogen (anoxia) has been assessed in *An. gambiae* (pupal stage) and *Culex quinquefasciatus* (pupal and adult stages) and it was reported that this did not have any, or very little, beneficial effects, and that higher doses were required to achieve the target sterility level [35, 36]. Another study showed that, in *Ae. aegypti* (pupae and adults), irradiation at 35, 70 and 100 Gy in nitrogen resulted in males equally competitive as non-irradiated males, compared to males irradiated in air that were not as competitive [63]. However, no known reports exist to date of the irradiation of mosquito pupae in oxygen depleted water and its effects on fertility and adult male biological quality.

Hallinan & Rai [63] demonstrated equal competitiveness in sterile and fertile *Ae. aegypti* males when adults were irradiated in nitrogen at 70 Gy and 100 Gy as compared to in air. At a lower dose of 35 Gy, the partially sterile (~ 60–70% sterile) males were three time more competitive than untreated males [63]. However, the samples were irradiated at the same dose in the two atmospheric conditions, meaning that the mosquitoes irradiated in nitrogen may have been more competitive, but are likely to have been less sterile, making these results of little use. Nevertheless, the authors’ suggestion that irradiation in nitrogen (anoxia) may be protective and resulting sterile males more competitive are likely to be true, as this was shown by Hooper [64] for *Ceratitis capitata* pupae, *Rodnius prolixus* [65] and *Bactrocera olea* (*Dacus olea*) [66] following irradiation in nitrogen *versus* air, where the irradiation dose was adjusted to achieve equal sterility levels and competitiveness was subsequently compared. Considering the existing information in other insect species, there is a chance that irradiation of mosquito pupae in oxygen depleted water or adults in nitrogen using optimal handling methods can improve sterile male competitiveness in the field. It is therefore, a priority to investigate this topic in further studies.

Among the mosquito species evaluated in this study, the greatest effects induced by hypoxia during irradiation was seen in *An. arabiensis*. This species may generally be more sensitive to oxygen effects during irradiation, or the radioprotective effects may have been highest in this species because the pupae depleted the water of DO the fastest, and thus were kept in lower DO levels for longer prior to irradiation than the two other species tested. It is also important to note that *An. arabiensis* was exposed the longest to radiation, as they require a higher dose for sterilization, which may partially explain why differences in IS were greater between the hypoxia and normoxia treatment groups.

In general, the doses needed for > 99% sterility in this study were relatively high compared to results reported elsewhere. Doses of 90, > 55 and > 120 Gy were needed to achieve over 99% IS in *Ae. aegypti*, *Ae. albopictus* and *An. arabiensis*, respectively, whereas former studies with the same strains have reported lower doses needed for this level of sterility: for *Ae. aegypti* (Brazil strain), between 35 and 50 Gy were needed using X-ray to reach 98–100% sterility (Maylen Gomez Pacheco, pers. comm.); for *Ae. albopictus* (Rimini strain) 35 Gy were sufficient using a cesium-137 source [67], and 40 Gy using X-ray [39, 40]; and for *An. arabiensis* (Dongola strain), 110 Gy were required using the same Gammacell 220 with a cobalt-60 source [23, 67]. One of the possible factors that could lead to these differences could be the oxygen content in the canister environment, or in the water during exposure.

## Conclusions

Other than the atmospheric conditions, other factors that may contribute to differences in the reported dose-response in pupae could include exposure temperature (which also effects respiration and DO concentrations in water), differences in dose-rate of the irradiators, differences in pupal age and thus radiosensitivity, changes in the strains’ biology over many generations of colonization and inbreeding or different genetic backgrounds among populations [39, 40]. Further research to look deeper into these possible factors is expected to aid in the development of standardized protocols for mosquito irradiation in the frame of the SIT to combat these important vector species.

## Data Availability

The datasets used and/or analyzed during the current study including all dosimetry reports are available from the corresponding author upon reasonable request.

## Abbreviations

AW-IPM: Area-Wide Integrated Pest Management
DO: dissolved oxygen
FAO: Food and Agriculture Organisation
IAEA: International Atomic Energy Agency
IPCL: Insect Pest Control Laboratory
IS: induced sterility
SIT: sterile insect technique
VAS: ventral air space
